# Assessing the genetic potential of a milk mid-infrared prediction of heat stress response in dairy cows using a temperature-humidity index–independent approach

**DOI:** 10.3168/jdsc.2026-1053

**Published:** 2026-06-11

**Authors:** P. Lemal, K. Wijnrocx, H. Soyeurt, M. Schroyen, N. Gengler

**Affiliations:** TERRA Teaching and Research Center, University of Liège, Gembloux Agro-Bio Tech (ULiège-GxABT), 5030 Gembloux, Belgium

## Abstract

Selection for heat tolerance remains challenging due to the difficulty of accessing reliable phenotypes at large scale. An alternative could be established using mid-infrared spectra, which are collected routinely through milk recording, and have already shown their value as proxies for a variety of phenotypes that are costly or difficult to measure. Recently, a first prediction of heat stress response in dairy cows based solely on milk mid-infrared spectra was developed. This prediction was obtained using models calibrated on surface body temperature and milk composition variations. Its potential as a detection tool was explored, but no genetic analyses has been performed. On this basis, the objectives of this study were to estimate the heritability of the predicted heat stress response, assess its genetic correlations with traits from the Walloon official genetic evaluation, and identify genomic regions associated with heat tolerance through a GWAS, all without using temperature-humidity index (THI) information. The estimated heritability (0.10) was low but sufficient to enable genetic selection and consistent with expectations for a heat stress–related trait. Also as expected, an antagonistic relationship between heat tolerance and milk production was observed, but its extent was notably reduced compared with traditional approaches. In addition, genetic correlations with other traits were neutral (fat yield) or favorable (protein yield, SCS, fertility, longevity). Concerning the GWAS, genomic regions and candidate genes previously associated with the response to heat stress were highlighted, as well as others related to energy balance maintenance. Overall, these results support the relevance of the prediction for the heat stress response as a new phenotype for heat tolerance selection that does not require any THI information. They also reinforce the importance of energy balance for dairy cows to cope with heat stress.

## Highlights

• Milk MIR-HS is heritable.
• The profile of genetic correlations between MIR-HS and routine traits is favorable.
• GWAS identified loci and genes related to thermotolerance and energy balance.
• MIR-HS shows potential as a phenotype for THI-independent heat tolerance selection.

## Graphical Abstract

**Figure:**
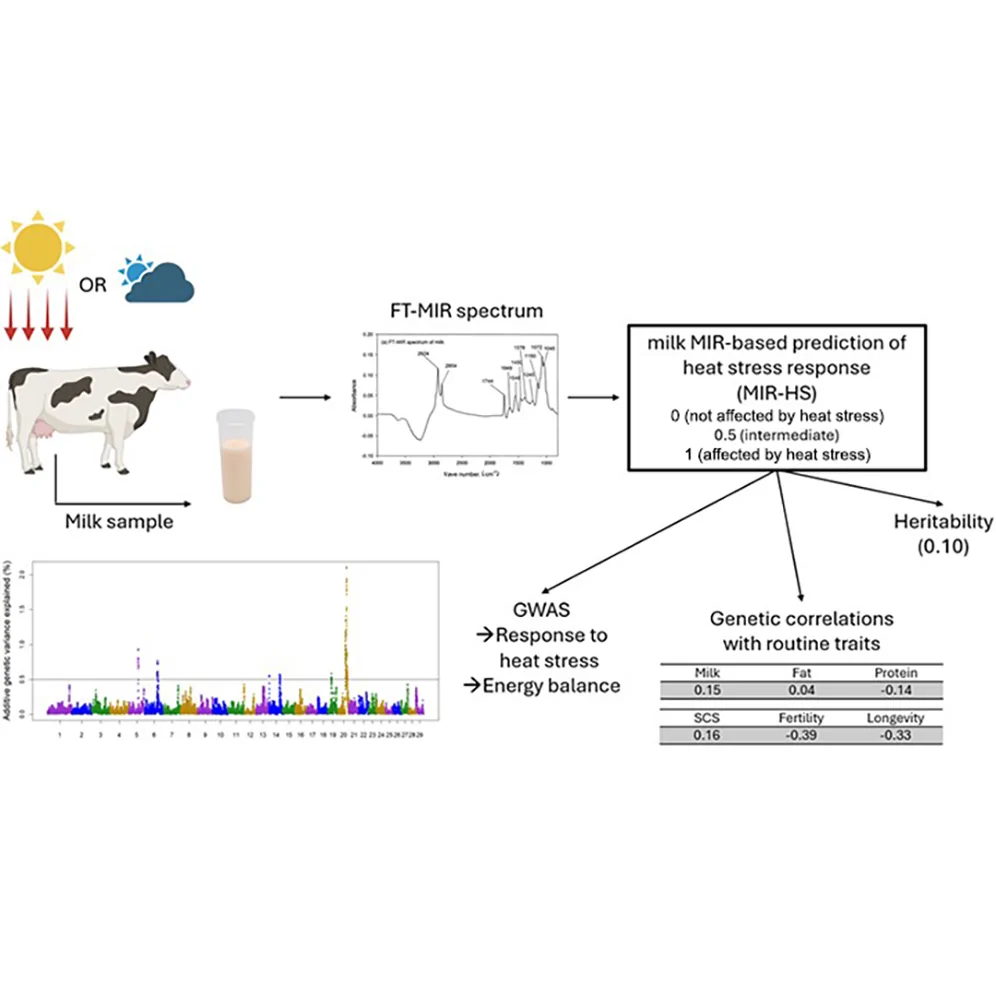
**Summary:** The milk mid-infrared (MIR)-based prediction of heat stress response (MIR-HS) is a new phenotype to detect heat stress effects in dairy cows through routine milk recording without extra costs. This study evaluated the relevance of MIR-HS as a potential indicator to select for heat tolerance using an approach that is totally independent of the temperature-humidity index (THI). The results showed that the trait is heritable and generally favorably correlated with traits included in official genetic evaluations. In addition, a genome-wide association study (GWAS) identified genomic regions and candidate genes related to heat stress response and energy balance. These findings highlight the potential of MIR-HS for improving heat tolerance in dairy cattle. This study also reinforces the central role of energy balance in the response to heat stress. Elements in upper left of the image created with BioRender.com.

A great limitation to select for heat tolerance in dairy cows is the difficulty to record reliable phenotypes at large scale (Misztal et al., 2025). Currently, the most common approaches use the decline in production with increasing temperature-humidity index (**THI**) to identify heat-sensitive animals, mainly because these data are easy to access (Carabaño et al., 2017). However, these approaches focus only on the production response to heat stress without considering other aspects, and they show a strong negative genetic correlation with overall milk production (Cheruiyot et al., 2022). In addition, these approaches rely entirely on THI or other environmental indicators that do not accurately represent the actual conditions experienced by individual cows (Hoffmann et al., 2020). An alternative method that better targets the heat stress response and its underlying mechanisms, fully independent of THI and still relying on inexpensive and widely available phenotypes, is the use of milk Fourier-transform mid-infrared (**FT-MIR**) spectra. Prediction equations for a variety of milk components have been developed based on FT-MIR spectra and some have already shown great potential as heat stress biomarkers (Hammami et al., 2015; Lemal et al., 2025). In addition, FT-MIR spectra are also used to predict more complex phenotypes that indirectly affect milk composition such as methane emissions, lameness, or feed intake (Grelet et al., 2021). Recently, a first proposition of milk mid-infrared (**MIR**)-based prediction of heat stress response (**MIR-HS**) in dairy cows, which relies only on spectra, was published (Lemal et al., 2026). This indicator was initially developed as a detection tool and no genetic analyses were performed. On this basis, the objectives of this study were to estimate its heritability and the genetic correlations with traits evaluated in official genetic evaluation, as well as to identify genomic regions of interest through a GWAS. These analyses will help both to validate the relevance of the prediction for genetic selection and to enhance our understanding of the heat stress response in dairy cows within a fully THI-independent approach.

To generate the dataset used in this study, standardized FT-MIR spectra were extracted from the official milk recording system of the Walloon region of Belgium from 2020 to 2023. Only records from Holstein cows with DIM between 5 and 365 and in herd test-day (**HTD**) with at least 10 available records were kept. A total of 1,002,263 spectra from 82,046 cows across 658 herds were obtained and the MIR-HS developed by Lemal et al. (2026) was applied. It combines 2 models: a partial least squares regression calibrated on surface body temperature, and a random forest model trained to classify cows according to their reactions to heat stress expressed through changes in surface body temperature and milk composition. This heat stress indicator takes 3 possible values (0, 0.5, or 1) with 0 an animal considered not affected by heat stress, 1 an animal considered affected by heat stress, and 0.5 an intermediate response. To restrict the dataset to HTD with a potential risk of heat stress while keeping the analyses fully independent of the THI, only HTD with an average MIR-HS of at least 0.1 were retained. This method of filtering ensures that the phenotype is actually expressed and maintains the initial objective of the indicator, which is to identify heat-stressed animals based on their individual responses rather than rely on environmental parameters such as THI that may not accurately reflect the conditions experienced by each cow. To remain consistent in the genetic analysis, reaction-norm methods requiring THI were thus not considered. As an alternative, the individual MIR-HS of the cows recorded during HTD with a potential risk of heat stress were directly used as phenotypes. Based on this, the final number of records included in the analysis was 171,687, recorded from 64,035 cows across 603 herds.

Even after selecting HTD suspected to be influenced by heat stress, most records were still classified as not affected (51%), followed by the intermediate class (33%), and the lowest frequency was obtained for the affected class (16%). To approximate as much as possible a normal distribution for the following genetic analysis, the predictions were transformed into Snell scores (Snell, 1964). Following the method detailed in Markey et al. (2025), Snell scores were computed from the cumulative frequencies associated with each class. For the central class, the score was obtained by transforming the cumulative frequencies of its lower and upper limits into values on the normal scale and averaging them. For the extreme classes, the Snell approximation was used, which adjusts the normal-scale value based on the cumulative frequency of the class.

To estimate variance components and GEBV the following repeatability model was used:

*y_jklmn_* = *HTD_j_* + *lact_k_* + *DIM_l_* + *a_m_* + *pe_m_* + *e_jklm_*,

where *y_jklmn_* is the MIR-HS transformed into Snell scores; *HTD_j_* is the categorical fixed effect for HTD of class *j*; *lact_k_* is the categorical fixed effect for the classes of lactation number *k* (4 classes: lactation 1, lactation 2, lactation 3, lactation 4+); *DIM_l_* is the categorical fixed effect for the classes of DIM *l* (classes of 5 DIM); *a_m_* is the additive genetic random effect for animal *m*; *pe_m_* is the permanent environment random effect for animal *m*, and *e_jklm_* is the residual.

This model was run using BLUPF90+ from the BLUPF90 family of programs (Misztal et al., 2014). The single-step GBLUP (**ssGBLUP**) approach was used to combine pedigree and genomic information. The pedigree contained 146,427 animals including 64,035 cows with phenotypes and 7,170 animals with genomic data for 28,442 SNPs after quality control using default parameters.

The heritability of the trait was calculated with the classical formula [h2=σa2/σa2(σa2+σpe2+σe2)(σa2+σpe2+σe2)]. A relatively low heritability value was estimated (0.10; Table 1). Although no similar trait has been reported in the literature, studies assessing the effect of heat stress on various traits have obtained a variety of heritability values. Dikmen et al. (2012) showed a heritability of 0.17 for rectal temperatures recorded during the summer. Ravagnolo and Misztal (2000) estimated heritability values ranging from 0.12 to 0.21 for milk, protein, and fat yields with a reaction-norm model on THI. In a subsequent study, the same authors (Ravagnolo and Misztal, 2002) also highlighted heritability values from 0.01 to 0.10 for the nonreturn rate using a similar method with univariate and bivariate models. More recently, Pook et al. (2025) estimated heritability values ranging from 0.05 to 0.12 for heat tolerance traits defined as residual slopes on THI for milk yield, fat, protein, lactose, and specific fatty acid concentrations. The heritability (0.10) obtained in this study is thus in the range expected for a heat stress–related trait.

**Table 1:** Heritability estimate for the prediction of heat stress response and genetic correlation estimates with routinely evaluated traits

Item	h^2^	Milk	Fat	Protein	SCS	Fertility	Longevity
Heat stress response	0.10	0.15	0.04	−0.14	0.16	−0.39	−0.33

To estimate genetic correlations with traits evaluated in routine, their GEBV and associated reliabilities were extracted from the official Walloon genetic evaluation system (Vanderick et al., 2022). Only animals (6,708) presenting GEBV with reliabilities of at least 0.5 for all the traits, including for the MIR-HS, were used in the following formula to estimate genetic correlations (Blanchard et al., 1983):

$$r_{i,j}=\frac{\sqrt{\left(\Sigma Rel_{i}\right)\times \left(\Sigma Rel_{j}\right)}}{\Sigma \left(Rel_{i}\times Rel_{j}\right)}\times r_{GEBVi,GEBVj},$$

where *r_i_*_,_*_j_* is the genetic correlation between trait *i* and trait *j*, *r_GEBVi_*_,_*_GEBVj_* is the correlation between GEBV of trait *i* and GEBV of trait *j*, *Rel_i_* is the reliability of the GEBV of trait *i*, and *Rel_j_* is the reliability of the GEBV of trait *j*.

Genetic correlation estimates are reported in Table 1. As expected, a positive genetic correlation was obtained between MIR-HS and milk yield (0.15), indicating that cows with higher milk yield tend to be the most affected by heat stress. In the literature, a similar trend is observed with negative genetic correlation between general milk production and milk heat tolerance effects (Aguilar et al., 2009; Carabaño et al., 2025). However, the correlation between milk yield and the predicted heat stress response is lower in magnitude than those previously reported. This suggests that selecting for low values of the MIR-HS may have a less detrimental effect on general milk yield than classical approaches used to assess heat stress. Moreover, a neutral genetic correlation was obtained with fat yield (0.04) and favorable genetic correlations were estimated with protein yield (−0.14), SCS (0.16), fertility (−0.39), and longevity (−0.33).

To identify genomic regions of interest, the single-step GWAS (**ssGWAS**) approach was used with the same model as described for the variance component and GEBV estimation. POSTGSF90 from the BLUPF90 family of programs (Misztal et al., 2014) was used to compute SNP effects by back-solving GEBV and to obtain the percentages of additive genetic variance explained by sliding windows of 20 SNPs. The Manhattan plot illustrating the additive genetic variance explained by these windows is shown in Figure 1. Nonoverlapping windows explaining at least 0.5% of the additive genetic variance were considered significant for a total of 12 regions located on chromosomes 5, 6, 14, 19, and 20 (Table 2).

**Figure 1:**
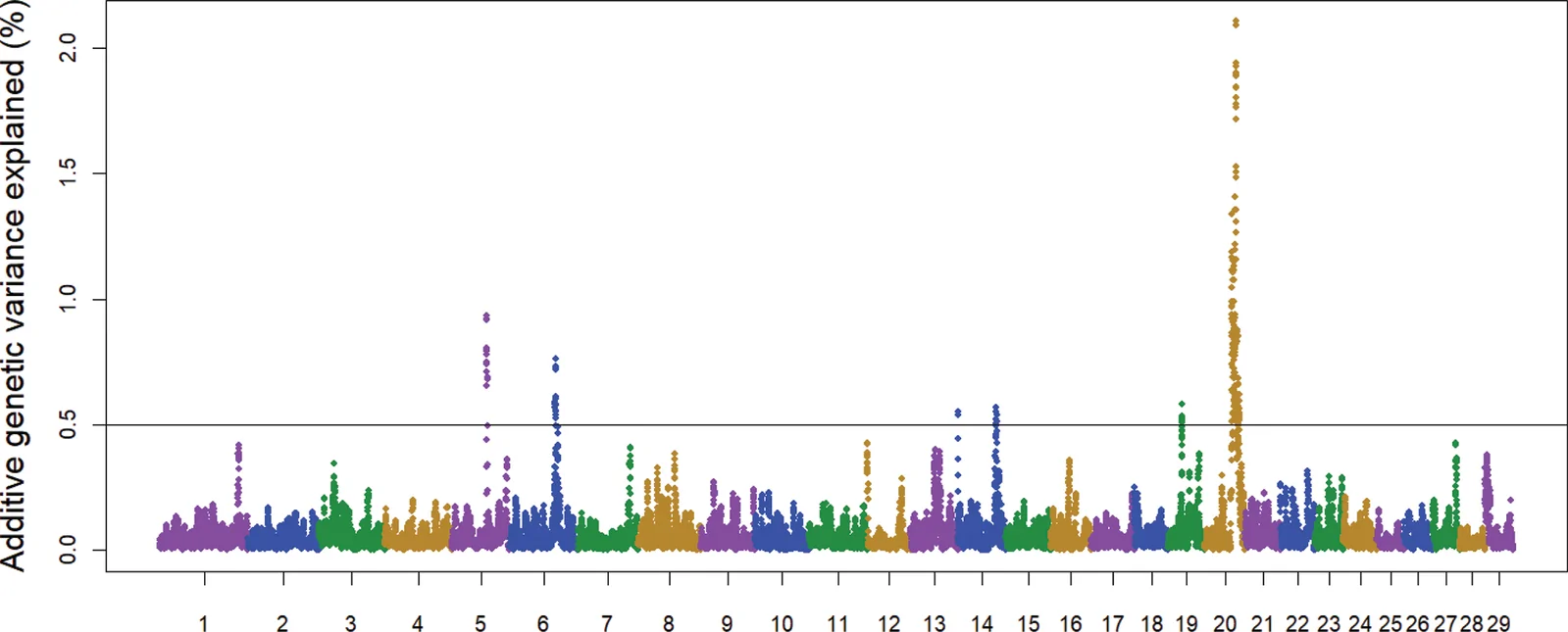
Manhattan plot representing the percentage of additive genetic variance explained by sliding windows of 20 SNPs across the 29 autosomal chromosomes for the prediction of heat stress response. Horizontal line: threshold of significance set at 0.5%.

**Table 2:** Candidate genes located within the 20-SNP windows explaining at least 0.5% of the additive genetic variance (σ_a_) for the prediction of heat stress response

Chromosome	Position (ARS-UCD1.2)^1^	% σ_a_ explained	Candidate genes
5	74,195,396–75,642,139	0.93	*ENSBTAG00000046392, ENSBTAG00000049297, ENSBTAG00000037799, ENSBTAG00000052884, ENSBTAG00000055135, ENSBTAG00000053500, ENSBTAG00000048740, ENSBTAG00000053144, ENSBTAG00000050417, ENSBTAG00000039524, ENSBTAG00000038652, APOL3, ENSBTAG00000012192, MYH9, ENSBTAG00000043161, TXN2, FOXRED2, EIF3D, ENSBTAG00000054427, CACNG2, IFT27, ENSBTAG00000008137, ENSBTAG00000048453, PVALB, NCF4, CSF2RB, ENSBTAG00000032114, TEX33, TST, MPST, KCTD17, TMPRSS6, IL2RB, C1QTNF6, SSTR3*
6	82,855,050–85,490,348	0.76	*CENPC, STAP1, UBA6, GNRHR, ENSBTAG00000038648, TMPRSS11D, TMPRSS11A, ENSBTAG00000025920, TMPRSS11F, ENSBTAG00000050708, TMPRSS11BNL, NAP1L1, TMPRSS11E, ENSBTAG00000044890, ENSBTAG00000044396, YTHDC1, ENSBTAG00000035726, ENSBTAG00000048013, ENSBTAG00000051713, ENSBTAG00000054846, ENSBTAG00000053068, ENSBTAG00000055078, UGT2B10, ENSBTAG00000049291, ENSBTAG00000045349, ENSBTAG00000003523, MGC152010, ENSBTAG00000052023, ENSBTAG00000053282, ENSBTAG00000053565, ENSBTAG00000015047, UGT2A1, SULT1B1, ENSBTAG00000038214, SULT1E1, CSN1S1, CSN2, HSTN*
14	279,949–1,472,679	0.55	*C14H8orf33, ZNF34, RPL8, ZNF7, COMMD5, ARHGAP39, ENSBTAG00000045478, C14H8orf82, LRRC24, LRRC14, ENSBTAG00000010276, MFSD3, GPT, PPP1R16A, FOXH1, KIFC2, CYHR1, TONSL, VPS28, ENSBTAG00000053637, SLC39A4, CPSF1, ADCK5, SLC52A2, FBXL6, TMEM249, SCRT1, DGAT1, HSF1, BOP1, SCX, MROH1, ENSBTAG00000054104, ENSBTAG00000044406, TSSK5, HGH1, WDR97, MAF1, ENSBTAG00000051469, SHARPIN, CYC1, GPAA1, EXOSC4, OPLAH, SMPD5, SPATC1, GRINA, PARP10, PLEC, ENSBTAG00000044263, EPPK1, NRBP2, PUF60, SCRIB, IQANK1, FAM83H, MAPK15, CCDC166, ZNF623, ENSBTAG00000052590, ENSBTAG00000052472, GFUS, PYCR3, TIGD5, EEF1D, NAPRT, MROH6, GSDMD, ZC3H3, MAFA, RHPN1, TOP1MT, ZNF696, GLI4, ENSBTAG00000054117, GPIHBP1, LY6H, ENSBTAG00000049400, LY6E*
14	63,925,324–65,629,300	0.57	*RNF19A, ENSBTAG00000050156, SPAG1, POLR2K, FBXO43, RGS22, COX6C, VPS13B, ENSBTAG00000042171, ENSBTAG00000037291, ENSBTAG00000030020, OSR2, STK3*
19	27,593,693–29,104,193	0.58	*CHD3, RNF227, KCNAB3, TRAPPC1, CNTROB, GUCY2D, ALOX15B, ALOX12B, ALOXE3, HES7, ENSBTAG00000043025, PER1, VAMP2, ENSBTAG00000053402, ENSBTAG00000043508, TMEM107, ENSBTAG00000051950, BORCS6, ENSBTAG00000049613, AURKB, CTC1, PFAS, RANGRF, SLC25A35, ARHGEF15, ODF4, ENSBTAG00000054068, KRBA2, RPL26, RNF222, ENSBTAG00000048507, NDEL1, MYH10, ENSBTAG00000043649, ENSBTAG00000043290, CCDC42, MFSD6L, PIK3R6, PIK3R5, ENSBTAG00000053249, NTN1, STX8, ENSBTAG00000006397, CFAP52, USP43, DHRS7C, GLP2R, RCVRN, GAS7, ENSBTAG00000043853*
20	49,918,452–51,156,345	1.34	*CDH12*
20	52,711,255–54,710,400	1.15	*CDH18, ENSBTAG00000043021, ENSBTAG00000054950, ENSBTAG00000052828*
20	55,232,425–56,792,004	0.90	*ENSBTAG00000045304, ENSBTAG00000053660, ENSBTAG00000012971, ENSBTAG00000049990, ENSBTAG00000054794, ENSBTAG00000051411, ENSBTAG00000049086, ENSBTAG00000048368, ENSBTAG00000053920, ENSBTAG00000055246, ENSBTAG00000049518, ENSBTAG00000053528, BASP1, ENSBTAG00000044221, ENSBTAG00000051157, MYO10, RETREG1, ZNF622*
20	57,277,558–58,586,804	1.41	*ENSBTAG00000048498, ENSBTAG00000045215, ANKH, OTULIN, OTULINL*
20	58,772,092–60,552,112	2.11	*TRIO, ENSBTAG00000054860, ENSBTAG00000049583, ENSBTAG00000043365, DNAH5, ENSBTAG00000053226*
20	61,310,753–62,365,196	0.67	*ENSBTAG00000049263, CTNND2, ENSBTAG00000045141, ENSBTAG00000042090*
20	62,537,740–63,521,064	0.85	*DAP, ANKRD33B, ROPN1L, MARCHF6, ENSBTAG00000052061, CMBL, CCT5, ATPSCKMT, ENSBTAG00000043875, ENSBTAG00000051332*

1 https://www.ncbi.nlm.nih.gov/datasets/genome/GCF_002263795.1/.

To identify genes within significant windows, the positions from the UMD3.1 genome assembly (provided by the Illumina BovineSNP50 BeadChip) were first converted to the more recent ARS-UCD1.2 assembly using Lift Genome Annotations (https://genome.ucsc.edu/cgi-bin/hgLiftOver). Then, gene annotations were extracted with the corresponding Ensembl BioMart tool (https://jul2023.archive.ensembl.org/biomart/martview/39ff4ab08932291bcbaf6ce04569fe34). The resulting list of genes is reported in Table 2, which includes genes previously identified in GWAS related to heat stress, such as *OTULINL* and *TRIO*, as well as genes associated with other relevant traits.

Indeed, another study focusing on heat stress also identified *OTULINL* and *TRIO* on chromosome 20 as candidate genes for the maximum surface temperature of the forehoof (Bang et al., 2025). A possible link between TRIO and heat stress may be through its ability to activate Rac1 (Bellanger et al., 1998) that is involved in heat shock responses (Gungor et al., 2014).

In a wider context, Sanchez et al. (2021) identified candidate genes for MIR-predicted milk mineral (Ca, P, Mg, K, and Na) and citrate content, and several of them overlapped with those detected in this study. These include *PPP1R16A* (citrate), *VPS28* (K), *FBXL6* (P), and *MROH1* (Ca) from the DGAT1 region on chromosome 14, as well as *ENSBTAG00000048498* (Ca, Mg, and citrate) and *ANKH* (K) on chromosome 20. Moreover, they also reported significant variants without annotated genes for citrate and Mg that are located on chromosome 20 within a significant window for the MIR-HS. Overall, the highest peak for Mg, citrate, and MIR-HS is located in a similar region of chromosome 20.

Because *DGAT1* is the most reported candidate gene for traits related to milk production (Khan et al., 2021; Bekele et al., 2023), it is thus expected to highlight the associated region for milk composition traits. However, *DGAT1* is located next to *HSF1* that is well known for its central role in cellular heat stress response (Collier et al., 2008), and SNPs in the *HSF1* gene have already been linked with heat tolerance in dairy cows (Li et al., 2011).

Similarly, on chromosome 20, the region including *ANKH* has already been highlighted in GWAS studies related to milk composition traits, especially to milk protein (Sanchez et al., 2017; Tiplady et al., 2021). Likewise, casein-related genes on chromosome 6 have been identified as candidate genes for MIR-HS.

However, the protein encoded by *ANKH* is also known to regulate extracellular pyrophosphate levels, an inhibitor of mineralization (Lopdell, 2023), and is thus an expected candidate for milk mineral composition. Because heat-stressed animals reduce their feed intake and lose more minerals through sweating (West, 1999), the regulation of mineral availability through genes like ANKH may affect the general response to heat stress.

In addition, the protein encoded by *ANKH* has been highlighted as being able to transport citrate (Szeri et al., 2020), which is also consistent with another GWAS focusing on MIR-precited milk citrate (Chen et al., 2024) that detected a similar peak on chromosome 20 and also proposed *ANKH* as candidate gene. The link between citrate transport and heat stress response could be through the regulation of energy balance. Indeed, milk citrate has been identified as an indicator of negative energy balance (Xu et al., 2020) and it has been proposed that detrimental effects of heat stress could be driven by negative energy balance, which results from the reduced feed intake and the increased energy requirement for maintenance (Bernabucci et al., 2010).

Supporting this concept, a GWAS on MIR-predicted milk BHB (Nayeri et al., 2019), another marker of negative energy balance, also showed several overlapping peaks with the MIR-HS. Common candidate genes were identified on chromosome 6 (*GNRHR*, *UGT2A1*, *CSN1S1*, *SULT1E1*, *YTHDC1*, *ENSBTAG00000038648*, *UGT2B10*, and *ENSBTAG00000048013*), on the DGAT1 region of chromosome 14 (*GRINA*, *WDR97*, *C14H8orf33*, *RHPN1*, *FOXH1*, *ARHGAP39*, *TONSL*, *NRBP2*, *SCRIB*, *LY6H*, *SHARPIN*, *MAF1*, *ZNF34*, *MROH1*, *SMPD5*, *OPLAH*, *ZNF7*, *HSF1*, *ZC3H3*, *DGAT1*, *CYHR1*, *PUF60*, *CCDC166*, and *ENSBTAG00000044406*), on another region of chromosome 14 (*RNF19A* and *RGS22*), and on chromosome 20 (*OTULINL*, *TRIO*, *ANKH*, *MYO10*, and *DNAH5*).

To further support the genetic relation between energy balance and MIR-HS, common candidate genes were also highlighted by a GWAS on MIR-predicted negative energy balance (*UGT2A1* and *SULT1B1* on chromosome 6; Hu et al., 2025) and on ketosis (*ZC3H3*, *MAFA*, *RHPN1*, *TOP1MT*, and *ZNF696* on DGAT1 region of chromosome 14 and *CDH12*, *DNAH5*, and *MYO10* on chromosome 20; Soares et al., 2021).

Since several GWAS cited in this study rely on MIR-predicted traits, the identified regions, especially the *DGAT1* region, may reflect milk composition rather than a specific physiological response to heat stress or energy balance. However, fluctuations in milk protein are known for both negative energy balance and heat stress response, potentially reflecting a metabolic shift. Therefore, the overlap between MIR-HS regions and regions identified in protein-related GWAS may reflect shared underlying biological mechanisms rather than MIR-specific artifacts. More generally, genes and genomic regions highlighted in GWAS require mechanistic validation to minimize interpretation bias. However, the high number of studies related to energy balance reporting regions similar to those identified for MIR-HS that include a study with a clinical ketosis trait independent of MIR (Soares et al., 2021) supports the idea already proposed in the literature that energy balance is important to cope with heat stress.

For the significant windows in this study that have not been reported in previous GWAS on heat stress or energy balance, the presence of relevant candidate genes indicates that potential connections to the heat stress response remain plausible. Indeed, among the genes found in these windows, *PER1* expression was modified in response to heat shock in zebrafish cells (Jerônimo et al., 2017), *ALOX15B* was involved in heat-induced apoptosis in pig Sertoli cells (Xue et al., 2022), *PIK3R5* was highlighted as important for heat stress adaptation in duck muscle tissues (Kim et al., 2017), and *TXN2* expression was reduced in the liver of heat-stressed broilers possibly leading to lower resistance to oxidative stress (Zhang et al., 2018).

Based on all these results, the MIR-HS developed by Lemal et al. (2026) presented a quite low heritability, as expected for a heat stress–related phenotype, but sufficient to enable selection. In addition, the genetic correlations with routinely evaluated traits were globally favorable with a low opposition between heat tolerance and milk production conversely to the most frequently used approaches. Finally, the identified genomic regions and the associated candidate genes were already highlighted in the literature for their implication in the heat stress response and in the maintenance of energy balance.

In conclusion, the genetic analyses performed in this study support the relevance of MIR-HS for heat tolerance selection within a fully THI-independent approach. Moreover, the genomic regions identified have also been reported in studies related to energy balance, supporting the hypothesis that it plays an important role in the heat stress response.
